# RIPOR2 Expression Decreased by HPV-16 E6 and E7 Oncoproteins: An Opportunity in the Search for Prognostic Biomarkers in Cervical Cancer

**DOI:** 10.3390/cells11233942

**Published:** 2022-12-06

**Authors:** Leslie Olmedo-Nieva, J. Omar Muñoz-Bello, Imelda Martínez-Ramírez, Antonio Daniel Martínez-Gutiérrez, Yunuen Ortiz-Pedraza, Claudia González-Espinosa, Vicente Madrid-Marina, Kirvis Torres-Poveda, Margarita Bahena-Roman, Marcela Lizano

**Affiliations:** 1 Unidad de Investigación Biomédica en Cáncer, Instituto Nacional de Cancerología, Avenida San Fernando 22, Sección XVI, Tlalpan, Mexico City 14080, Mexico; 2 Laboratorio de Genómica, Instituto Nacional de Cancerología, Tlalpan, Mexico City 14080, Mexico; 3 Departamento de Farmacobiología, Centro de Investigación y de Estudios Avanzados, Unidad Sede Sur, Calzada de los Tenorios 235, Granjas Coapa, Tlalpan, Mexico City 14330, Mexico; 4 Centro de Investigación en Enfermedades Infecciosas, Instituto Nacional de Salud Pública, Cuernavaca, Morelos 62100, Mexico; 5 Departamento de Medicina Genómica y Toxicología Ambiental, Instituto de Investigaciones Biomédicas, Universidad Nacional Autónoma de México, Circuito Exterior S/N, Ciudad Universitaria, Mexico City 04510, Mexico

**Keywords:** RIPOR2, prognostic biomarker, HPV, cervical cancer, HPV-16 E6 and E7

## Abstract

High-risk human papillomavirus (HPV) infection is the main risk factor for cervical cancer (CC) development, where the continuous expression of E6 and E7 oncoproteins maintain the malignant phenotype. In Mexico, around 70% of CC cases are diagnosed in advanced stages, impacting the survival of patients. The aim of this work was to identify biomarkers affected by HPV-16 E6 and E7 oncoproteins that impact the prognosis of CC patients. Expression profiles dependent on E6 and E7 oncoproteins, as well as their relationship with biological processes and cellular signaling pathways, were analyzed in CC cells. A comparison among expression profiles of E6- and E7-expressing cells and that from a CC cohort obtained from The Cancer Genome Atlas (TCGA) demonstrated that the expression of 13 genes impacts the overall survival (OS). A multivariate analysis revealed that the downregulated expression of RIPOR2 was strongly associated with a worse OS. RIPOR2, including its transcriptional variants, were overwhelmingly depleted in E6- and E7-expressing cells. Finally, in a Mexican cohort, it was found that in premalignant cervical lesions, RIPOR2 expression decreases as the lesions progress; meanwhile, decreased RIPOR2 expression was also associated with a worse OS in CC patients.

## 1. Introduction

Cervical cancer (CC) ranks fourth in cancer mortality in women worldwide, while, in Mexico, it ranks second. This neoplasia continues to be a public health problem, since, in the last decade, there has been a considerable increase from 3357 cervical cancer deaths estimated in 2012 to 4335 cases in 2020 [1]. The main risk factor attributed to the development of CC is a persistent infection with high-risk (HR) human papillomaviruses (HPV), whose genome has been found in most of the cervical cancer cases (up to 90%) [2]. The most prevalent viral type in cervical cancer is HPV-16, which is found in 50% of all cases [3]. 

The oncogenicity of HPV lies mainly in the continuous expression of E6 and E7 oncogenes, whose protein products interact with different cellular proteins that promote cancer-associated processes such as proliferation, migration, invasion, the inhibition of apoptosis, and the evasion of the immune response, among others [4]. One of the most studied functions of viral oncoproteins is the degradation of tumor suppressor proteins. E6 interacts with p53 and with the ubiquitin ligase E6AP, promoting the degradation of p53 through the proteasome, and this event allows the inhibition of apoptosis, the promotion of genomic instability, and the accumulation of mutations [5,6]. The E7 protein interacts with pRb and with the ubiquitin ligase Cullin2, favoring pRb proteasomal degradation. This event promotes the translocation of the E2F transcriptional factor to the nucleus and the transcription of genes related to G1-to-S-phase transition, promoting the continuity of the cell cycle [7]. 

In developing countries, such as Mexico, a high proportion of CC cases are diagnosed in advanced clinical stages, resulting in lower survival and a high mortality rate [8], which is largely due to the lack of effective cervical cancer screening programs. In Mexico, more than 70% of cervical cancer patients are detected in locally advanced or advanced stages [9], while the overall survival (OS) worsens as the clinical stage progresses [10]. Disease characteristics related to clinical stages, such as tumor size, lymph node infiltration, and distant metastasis, are related to patient survival; however, not all patients with the same clinical stage have the same outcome. Therefore, some studies have focused on searching for molecules that can predict patient survival. In this regard, some proteins have been proposed as prognostic biomarkers for CC, including the increased of Ki-67/MIB-1 protein levels [11], glucose-6-phosphatase catalytic subunit (G6PC) [12], and serine/arginine-rich protein-specific kinase 1 (SRPK1) [13], which are related with worse survival, while the high levels of Galectin 9 [14] correlate with a better prognosis in CC patients. Moreover, through the analysis of transcriptional profiles derived from genomic databases of CC patients, genes related to OS have been identified [15,16]. For instance, the high expression of BRCA1 [17] is associated with better OS, while high levels of VEGF165 transcript have been associated with worse disease-free survival [18] in CC patients. Alterations of non-coding RNAs have also been proposed as prognostic biomarkers in CC [19,20,21].

Since viral oncoproteins are responsible for maintaining the malignant phenotype, strategies aimed at finding new HPV-dependent biomarkers have been explored. The detection of HPV DNA and mRNA has been used for determining the risk of progression to cancer and as prognostic biomarkers. E6 and E7 transcripts have been shown to have higher specificity compared to HPV DNA positivity [22,23] and a higher positive predictive value of progressing to high cervical squamous intraepithelial lesions (HSIL) or cancer. Furthermore, it has been demonstrated that the presence and levels of E6 transcripts increase the risk of progression to cancer [24]. In cervical cancer, high expression of E6 oncogene and its isoform E6* are associated with poor overall survival [25]. Furthermore, the use of HPV mRNA as a molecular marker for cervical cancer metastatic spread tumor has been proposed [26,27]. In the sentinel node (SLN) of patients free of lymph node metastases, it was demonstrated that the presence of HPV mRNA has a prognostic value independent of tumor size, where recurrence-free survival was significantly longer for patients whose SLN was negative for HPV mRNA [27]. Genetic expression profiles dependent on viral oncogenes in CC offer a novel alternative in the search for biomarkers with prognostic value. A more precise classification of CC cases according to molecular profiles, considering viral oncogene expression would be useful to identify patients with more aggressive tumors. In addition, this information may identify targetable molecules as novel therapeutic potential options for patients with cervical cancer. The aim of this study was to identify molecules with potential as prognostic biomarkers, deregulated by HPV-16 E6 and E7 oncogenes that may impact the clinical outcome of patients with cervical cancer. Results showed that several transcripts were found to be altered by the E6 and E7 oncoproteins both in a cell model and in cervical cancer, where the decreased expression of RIPOR2 (RHO 2 family-interacting cell polarization regulators) was associated with poor OS, regardless of clinical stage. These findings position RIPOR2 as a potential prognostic biomarker in cervical cancer.

## 2. Materials and Methods

### 2.1. Cell Lines and Culture

Cervical cancer cell lines C-33 A, SiHa. and Ca Ski were purchased from ATCC (Manassas, VA, USA) and maintained at 37 °C with 5% CO_2_. SiHa and C-33 A cells were grown in Dulbecco’s modified Eagle’s medium (DMEM) and Ca Ski cells in Roswell Park Memorial Institute (RPMI) medium, all supplemented with 10% of fetal bovine serum (FBS). C-33 A cells were stably transfected with the indicated plasmids using Lipofectamine reagent (Invitrogen, Waltham, MA, USA) according to the manufacturer’s instructions, and selection was performed with 2 g/L of G418 (ChemCruz Bio, Dallas, TX, USA). The isolated C33-EV, C33-E616, and C33-E716 clones were used for specified experiments.

### 2.2. Plasmids

HPV-16 E6 and E7 Open Reading Frames (ORFs) were amplified from Ca Ski DNA using Polymerase Chain Reaction (PCR). Viral sequences, including an HA tag sequence, were amplified with specific primers (Supplementary Table S1) and cloned into the p3x-FLAG CMV.10 expression vector (Sigma, Burlington, MA, USA). Constructions were verified by DNA-sequencing. Finally, the plasmids named as empty vector p3x-FLAG (EV), p3x-FLAG-HA-E616 (E616), and p3x-FLAG-HA-E716 (E716) were used for the transfections of C-33 A cells to obtain stably transfected cells C33-EV, C33-E616, and C33-E716.

### 2.3. Western Blotting 

C33-EV, C33-E616, and C33-E716 cells were cultured in 60 mm dishes and after 24 h lysed using 300 µL of RIPA buffer (100 mM Tris pH 8.0, 50 mM NaCl2, 0.5% Nonidet P-40, and protease inhibitor cocktail (Roche, Basel, CH)). A total of 20 μg of cell protein extracts were analyzed by SDS-PAGE gels (10–12%) and blotted onto a 0.22 µm nitrocellulose membrane (Bio-Rad, Hercules, CA, USA). Membranes were blocked with 10% skimmed milk in TBS-0.1% Tween 20 for 1 h at room temperature, followed by incubation with anti-HA (Cell Signaling, Danvers, MA, USA) and anti-H4 (Cell Signaling, Danvers, MA, USA) primary antibodies diluted 1:1000 and 1:20,000, respectively. After washing three times with TBS-0.1% Tween 20, membranes were incubated with HRP-conjugated secondary anti-mouse antibody (Santa Cruz, Bio., Dallas, TX, USA) in a dilution 1:10,000. Proteins were visualized utilizing the Clarity™ Western ECL Substrate (Bio-Rad, Hercules, CA, USA), according to the manufacturer’s instructions. Then, membranes were visualized and analyzed in the iBright FL1500 imagining system (Invitrogen, Waltham, MA, USA).

### 2.4. Immunofluorescence Staining

Stable C-33 A cells were seeded over cover slides in 6-well plates. After 24 h, cells were fixed using 3.7% paraformaldehyde/PBS for 10 min and permeabilized with PBS-0.1% Triton-X100. Then, cells were blocked with a 0.3% BSA solution and incubated overnight at 4 °C with anti-HA antibody (Cell Signaling, Danvers, MA, USA) diluted 1:50. Cells were extensively washed with PBS and later incubated with anti-rabbit antibody conjugated to Alexa-488 (Invitrogen, Waltham, MA, USA) diluted 1:700. Slides were washed and mounted with Vectashield antifade mounting medium with DAPI (Vector laboratories, Burlingame, CA, USA). Cells were analyzed with EVOS FL fluorescence Microscope (Invitrogen, Waltham, MA, USA).

### 2.5. RNA Sequencing and Data Analysis

Total RNA was extracted from C33-EV, C33-E616, and C33-E716 cells using the RNeasy mini kit (Qiagen, Hilden, DE), according to the manufacturer’s instructions. Three independent experiments of each condition were performed to ensure reproducibility. RNA integrity was verified through the Bioanalyzer 2100 system (Agilent, Santa Clara, CA, USA). RNA library preparation and sequencing was carried out by Novogene Bioinformatics Technology Co., Ltd. (Sacramento, CA, USA). Sequencing results were mapped zwith the reference human genome GRCh38, and the differential expression analysis was obtained comparing groups C33-EV vs. C33-E616 and C33-EV vs. C33-E716 using the DESeq2 R package (1.16.1). Genes with adjusted *p*-value < 0.05 were considered as differentially expressed. Enrichment analysis of differentially expressed genes was implemented by the clusterProfiler R package for Gene Ontology (GO) [28] Encyclopedia of Genes and Genomes (KEGG) pathways [29] and Reactome [30]. Terms with corrected *p* value < 0.05 were considered significantly enriched by differentially expressed genes. 

### 2.6. TCGA Analysis

Data from 309 cervical samples from the TCGA project were downloaded using the Bioconductor package TCGABiolinks [31]. Differential expression analysis was performed between normal tissue and tumoral samples using the DESeq2 package [32] and considering those transcripts with an *p*-adj < 0.05 as statistically significant.

### 2.7. Real-Time Quantitative PCR 

Cells were seeded in 60 mm culture dishes, and 24 h after, total RNA extraction was performed using the RNeasy mini kit (Qiagen, Hilden, DE). The isolated RNA was treated with the DNase-Free DNA removal kit (Ambion, Austin, TX, USA), and 1000 µg of RNA was reverse-transcribed with random hexamers utilizing the GeneAmp RNA PCR Core Kit (Applied Biosystems, Waltham, MA, USA). The primers used for amplification of the different targets analyzed are contained in Supplementary Table S1. Maxima SYBR green/ROX qPCR Master Mix (2×) (Thermo Scientific, Waltham, MA, USA) was used for qPCR reaction. The results are presented as relative quantification using the ΔΔCt method.

### 2.8. Cervical Samples

A cohort of samples from Mexican patients with normal and premalignant lesions of the uterine cervix was tested for RIPOR2 expression, formed by 17 normal HPV-negative cervical samples, 7 normal HPV-positive cervical samples, 20 low-grade, and 15 high-grade cervical premalignant lesions, kindly provided by the Instituto Nacional de Salud Pública (INSP). In addition, 19 cervical cancer samples from the Tumor BioBank from the Instituto Nacional de Cancerología of Mexico City (INCan) were included. The protocol was revised and accepted on February 2017, by the Scientific and Ethical committees of INCan Ref. (017/007/IBI)(CEI/1144/17). All patients whose samples were utilized in this study agreed and signed the informed consent. 

### 2.9. Statistical Analysis

Data showing the effects of HPV-16 E6 and E7 proteins on RIPOR2 transcript levels are presented as the mean ± SD. Analyses were performed using GraphPad Prism 5 software; *p*-value was calculated by Student’s *t*-test and significant differences were accepted when *p* < 0.05, as indicated. To assess RIPOR2 expression in premalignant lesions compared to normal cervical samples, the statistical analysis was performed using Mann–Whitney *U* statistical test. For the survival analysis, clinical and follow-up data from the 309 cervical samples from the TCGA was obtained with the TCGABiolinks package. For each gene, patients were divided into two groups depending on the median expression as high or low. The overall survival of patients depending on analyzed gene was calculated using the Kaplan–Meier estimator. Comparison of the survival curves for both groups was performed using the log–rank test. Next, we performed Univariate and Multivariate Cox proportional hazard regressions using the R survival package. We considered a *p*-value < 0.05 as significant.

## 3. Results

### 3.1. HPV-16 E6 and E7 Oncoproteins Differentially Modify Transcriptome of Cervical Cancer Cells

**Figure 1:**
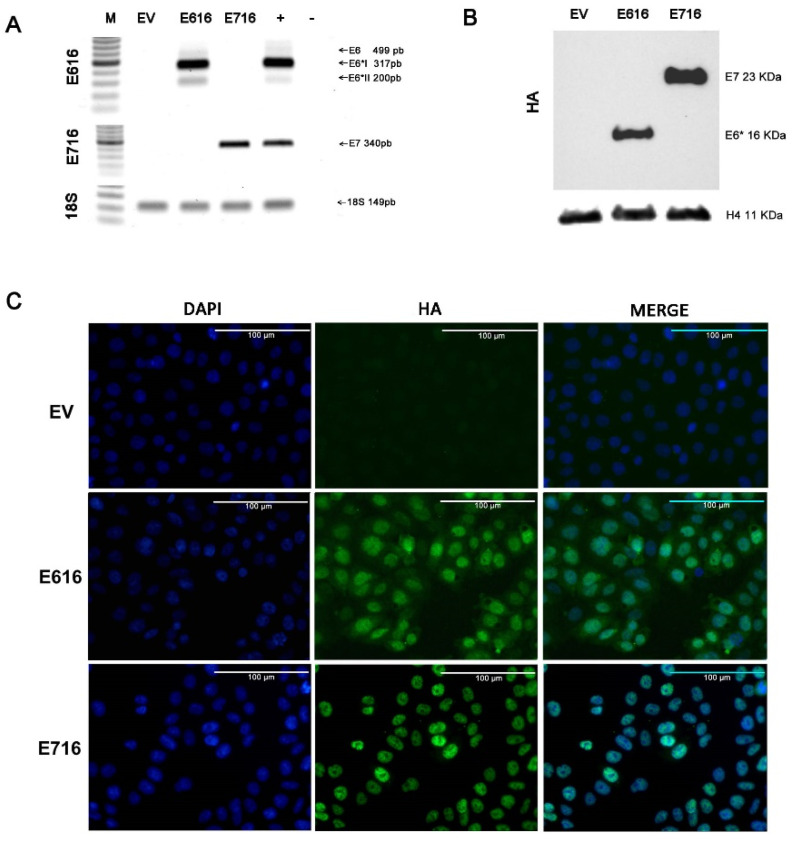
Stable expression of E616 and E716 in C-33 A cells: (**A**) RT-PCR showing the expression of E6, E6*I, and E6*II mRNA in C33-E616 cells, as well as the E7 mRNA in C33-E716 cells. 18S rRNA expression was used as a control. (**B**) Detection of HA-tagged E6 and E7 proteins by WB in stable C-33 A cell lines using HA antibody. H4 protein was used as the loading control; (**C**) Immunofluorescence staining using DAPI nuclear detection (blue) and anti-HA primary antibody to detect E6 and E7 oncoproteins (green). A representative image of each experiment is shown. Scale bar represents 100 µm long.

To analyze the effect of HPV-16 E6 and E7 oncoproteins on cell gene expression profiles, a model of C-33 A cells stably transfected with vectors expressing E616 or E716 oncoproteins was generated, while cells harboring empty vector (EV) were used as a negative control. The expression of the E6 and E7 transcripts was assessed by RT-PCR in the three cell lines (Figure 1A). As expected, the expression of full-length E6 and its small isoforms E6*I and E6*II were detected in the C33-E616 cell line, as it has been reported in HPV-positive cells [33,34]. On the other hand, E716-containing cells (C33-E716) only expressed E7 transcripts. The presence of the oncoproteins was also evaluated by immunoblot (Figure 1B). It is worth noting that protein levels of E6 full-length were hardly perceptible, even with long immunodetection exposure (data not shown), while the small isoform E6* is highly abundant. Meanwhile, the E7 protein was clearly detected in stably transfected cells. The immunofluorescence analysis showed that E6 and E7 were localized mainly at the nucleus and were present in all transfected cells, confirming that the model with a stable expression of the oncoproteins was successful (Figure 1C). 

**Figure 2:**
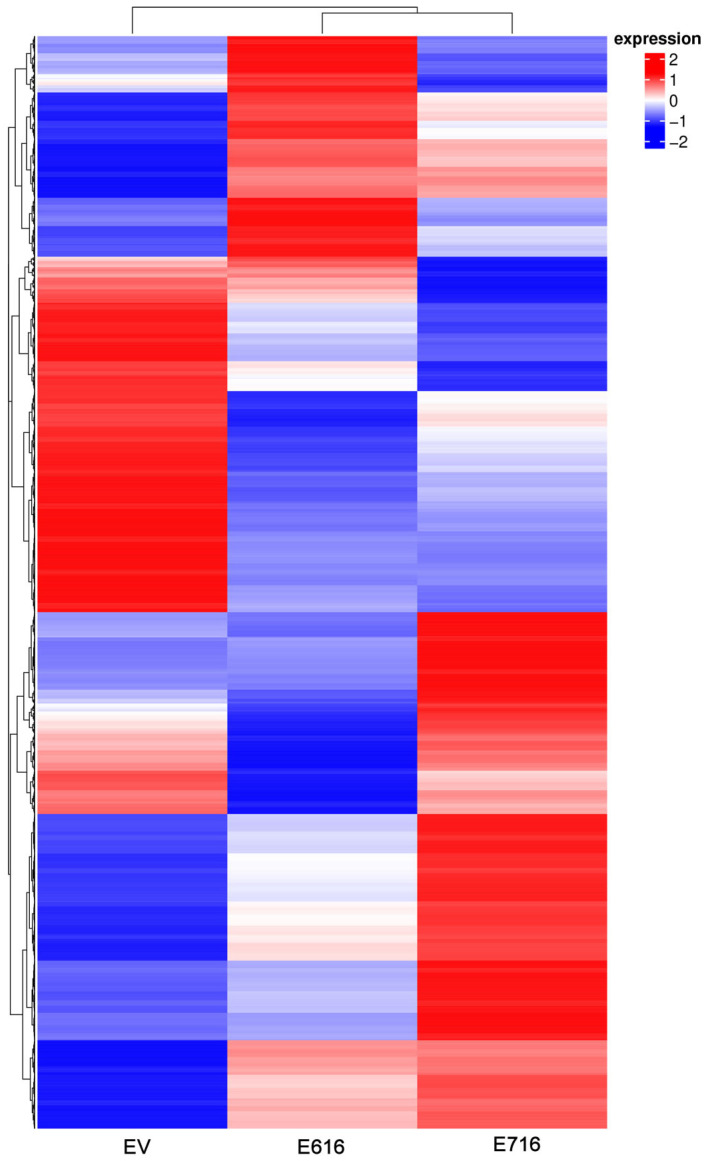
Gene expression patterns exhibited by C33EV, -E616, and -E716 cells. Heatmap showing the differential gene expression in Log2(FPKM+1) in the three cell groups in the columns (EV, E616, and E716). Each row represents the expression of a gene. Red color indicates increased expression levels and blue, decreased expression, while white means no significant change or the absence of data. Hierarchical clustering is shown at the top of the figure according to the transcriptional patterns of the groups (EV, E616, and E716), revealing that cells expressing the oncoproteins are closer than those with the empty vector. At the left, the clustering for differential gene expression is depicted.

To identify gene expression profiles associated with the expression of E6 and E7 oncogenes, a mRNA massive sequencing analysis was performed in C-33 A stably transfected cells (E616, E716, or EV). Evident differential expression patterns were exhibited in E6- and E7-expressing cells when compared to the control group, as depicted in the heatmap of Figure 2. Differentially expressed genes are shown in Supplementary Tables S2 and S3.

**Figure 3:**
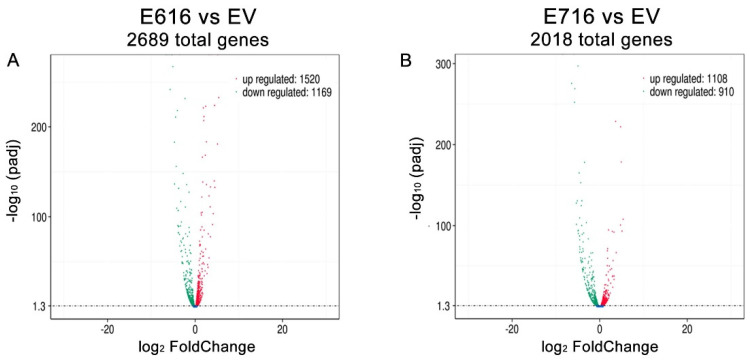
E616 and E716 differentially affected gene expression in C-33 A cells. Volcano plot illustrating the genes that were significantly (*p*-adj < 0.05) deregulated in: (**A**) C33-E616 cells and (**B**) C33-E716 cells, compared with the EV control. The upregulated genes are depicted in red color and the downregulated ones in green.

A differential gene expression analysis was performed by comparing the gene expression levels (Log2 FC) in C33-E616 and C33-E716 cells in relation to C33-EV (Tables S2 and S3). A total of 2689 genes were found significantly differentially expressed (*p*-adj < 0.05) in the presence of E6. From those genes, 1520 were upregulated, while 1169 were downregulated (Figure 3A). Similarly, when comparing C33-E716 cells with C33-EV, 2018 genes were significantly deregulated (*p*-adj < 0.05), of which 1108 were upregulated and 910 were downregulated (Figure 3B). 

### 3.2. Cellular Processes and Signaling Pathways Modified by E6 and E7

An enrichment analysis was performed to identify pathways and biological functions significantly affected by E616 and E716. For this purpose, information from three different databases, including Gene Ontology (GO), Kyoto Encyclopedia of Genes and Genomes (KEGG), and Reactome database, was used.

**Figure 4:**
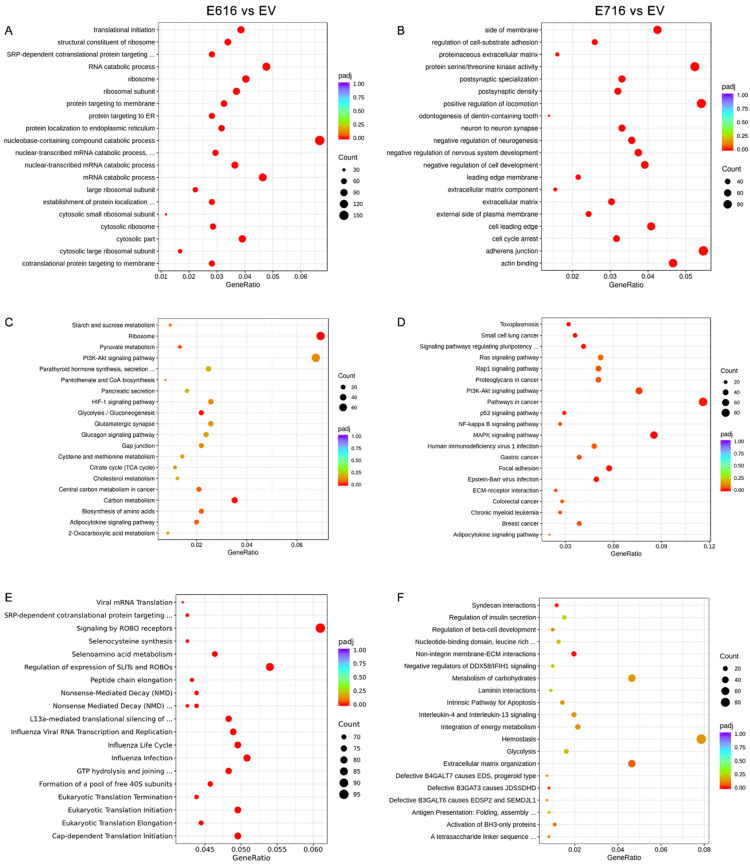
Enrichment analysis of differentially expressed genes (DEGs) in cells containing E616 and E716 oncoproteins. Dot plots of the 20 biological functions or pathways more significantly related with the DEGs modulated by E616 and E716 are depicted. Enrichment analysis was performed using data from GO for (**A**) E6- and (**B**) E7-expressing cells; while KEGG analysis exhibited cellular pathways affected in (**C**) E6- and (**D**) E7-containing cells. Reactome analysis showed processes associated with (**E**) E6 and (**F**) E7 expression. Significantly deregulated processes (*p*-adj < 0.05) were depicted in red color. Count means the number of genes assigned to a term. GeneRatio refers to the number of observed genes (DEGs) divided by the number of expected genes related to each category.

When evaluating the sets of genes deregulated by E616, the GO enrichment analysis demonstrated that processes of nucleobase-containing compounds of catabolism, ribosomes, and translation were mostly affected (Figure 4A). Furthermore, a KEGG analysis showed that the top deregulated pathways included ribosomes, carbon metabolism, and glycolysis/gluconeogenesis (Figure 4C), and the Reactome analysis showed that processes related with ROBO proteins and translation are also deregulated by E616 (Figure 4E).

Regarding those processes altered by E716, the GO analysis demonstrated that the positive regulation of locomotion, adherens junctions, protein serine/threonine kinase activity, and actin binding are among the most deregulated processes (Figure 4B). Meanwhile, the KEGG analysis showed that E716 deregulated genes involved in the pathways in cancer, including MAPK, PI3K/Akt, NF-kB, and Ras signaling, among others (Figure 4D). Furthermore, the most significant processes revealed by the Reactome analysis were those related to syndecan interactions and non-integrin membrane–extracellular matrix interactions (Figure 4F).

### 3.3. E616 and E716 Regulated Genes Involved in Overall Survival of Cervical Cancer Patients

**Figure 5:**
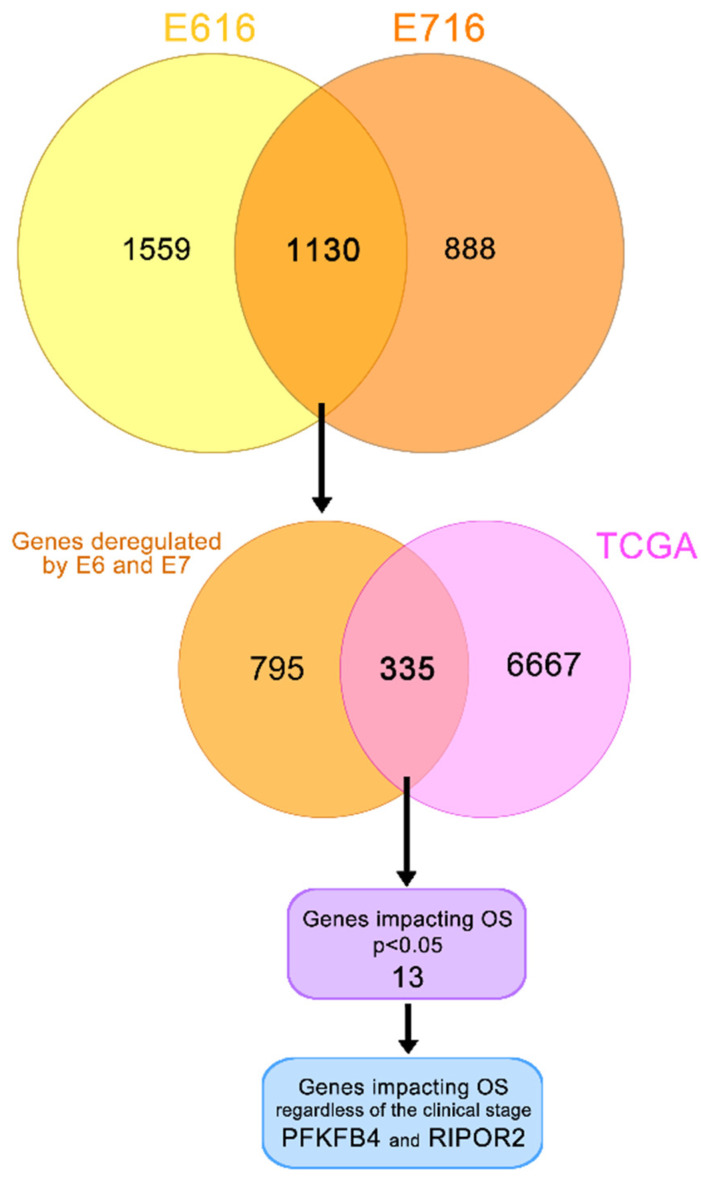
Genes deregulated in cervical cancer and in E6- and E7-expressing cells. Yellow/orange Venn diagram shows the genes deregulated by E616 and E716 in C-33 A stably transfected cells; the intersection of this diagram refers to the 1130 genes significantly modulated by both viral oncoproteins. Orange/pink Venn diagram intersects 335 genes modulated in CC according to the data obtained from TCGA and by the E6 and E7 viral oncoproteins. From these data, a univariate analysis showed 13 genes significantly affecting the OS (*p* < 0.05). A multivariate analysis demonstrated that PFKFB4 and RIPOR2 genes affected the OS independently of the clinical stage (*p* < 0.05).

To determine genes affected by both oncoproteins, a Venn diagram was constructed (Figure 5). The results indicated that 1130 genes were deregulated by both E616 and E716 in C-33 A stable cell lines. Since E6 and E7 are constitutively overexpressed in CC, the aim of this study was to analyze those genes that were affected by both oncoproteins. Bioinformatic analyses derived from a TCGA cohort revealed differentially expressed genes in CC compared with normal tissue in data obtained from 309 cervical cancer patients. The results demonstrated that 6667 genes were significantly (*p* < 0.05) deregulated in CC. From those, 335 genes that were deregulated in CC patient samples, as well as in C33-E616 and C33-E716 cells. 

A univariate Cox regression analysis exposed that 13 of these 335 genes significantly (*p* < 0.05) affected the OS in CC patients, as shown in Table 1. Since the OS is also affected by the clinical stage, the independence of the clinical stage was analyzed through a multivariate analysis, which demonstrated that the expression of two genes act as independent predictors of the OS; interestingly, a high RIPOR2 expression increases the OS (HR = 1.8, CI 1.00–3.25, *p* = 0.048), while a high expression of PFKFB4 decreases the OS (HR = 0.50, CI 0.27–0.93, *p* = 0.029) (Table 1).

**Figure 6:**
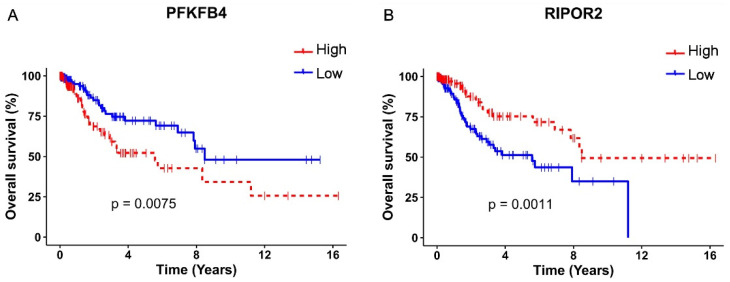
Kaplan–Meier OS analysis according to PFKFB4 and RIPOR2 expressions. Differences in OS of CC patients according to high or low expression of: (**A**) PFKFB4 (*p* = 0.0075) and (**B**) RIPOR2 (*p* = 0.0011). Low mRNA levels are represented with blue lines and high levels with red lines.

The survival analysis and Kaplan–Meyer curves were performed taking into consideration the high or low expression of PFKFB4 and RIPOR2, according to the median expression levels, in TCGA cervical cancer samples. As depicted in Figure 6A, a high expression of PFKFB4 was found associated with unfavorable OS (*p* = 0.0075), evidenced by the decrease in the median survival from 8.48 years in patients with a low expression of PFKFB4 to 5.57 years in patients with a high expression. Contrariwise, a high expression of RIPOR2 exhibited a protector effect (*p* = 0.0011) (Figure 6B), since patients with high expression showed a median survival of 8.48 years compared to 5.57 years in patients who expressed low levels of RIPOR2. The obtained results evidence that RIPOR2 and PFKFB4 are deregulated in CC patients and in C33-E616 and C33-E7 CC cell lines, suggesting that their modulation in this cancer type is partially mediated by E6 and E7 oncoproteins. 

### 3.4. PFKFB4 and RIPOR2 Transcripts Are Affected by E6 and E7 in Cervical Cancer Cells

**Figure 7:**
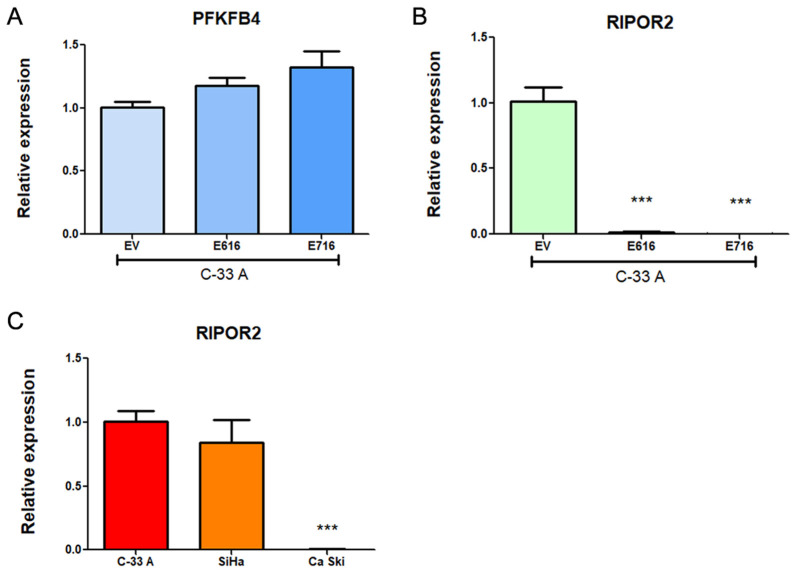
Expression of PFKFB4 and RIPOR2 in CC cell lines. Results obtained by RT-qPCR in C33-E616 and C33-E716 compared to C33-EV for: (**A**) PFKFB4 mRNA levels; (**B**) RIPOR2 mRNA levels; and (**C**) RIPOR2 mRNA levels in CC cell lines C-33 A, SiHa, and Ca Ski. Each graph is a representative experiment from three independently performed. Statistics was performed using GraphPad prism, mean ± SD, Student’s *t*-test, *** *p* < 0.0001.

To validate the results obtained in the RNAseq analysis, transcript levels of PFKFB4 and RIPOR2 were analyzed in C-33 A E6- and E7-expressing cells in relation to EV cells through RT-qPCR. Surprisingly, as shown in Figure 7A, a trend for increased expression of PFKFB4 was observed in E6- and E7-expressing cells, although no statistical changes were obtained. In contrast, RIPOR2 levels were overwhelmingly ablated by E616 and E716 (*p* < 0.0001) (Figure 7B). These results were comparable with those obtained for RIPOR2 in the RNAseq analysis, where its expression levels were Log2FC–2.622 (*p* = 7.16–39) and Log2FC–3.839 (*p* = 4.32–44) for cells expressing E616 and E716, respectively. Those data corroborate the effect of both oncoproteins in the decrease of RIPOR2 mRNA levels in the CC cell line C-33 A. Furthermore, the expression of RIPOR2 was analyzed in CC cell lines harboring HPV-16 sequences. As shown in Figure 7C, SiHa cells did not exhibit significant differences in RIPOR2 expression levels in relation to C-33 A cells. In contrast, Ca Ski cells practically did not express RIPOR2. These results may be partially explained by the differences in HPV copy number which may influence the RIPOR2 expression levels, since Ca Ski cells harbor 500 HPV viral copies and SiHa cells contain 1–2 copies [35]. 

### 3.5. HPV-16 E6 and E7 Oncoproteins Decrease the Levels of Six Transcriptional Variants of RIPOR2 in C-33 A Cells

According to the National Center for Biotechnology Information (NCBI) [36], there are at least seven RIPOR2 transcriptional variants, which code for six different RIPOR2 protein isoforms (Table 2). Therefore, we became interested in investigating the impact of HPV oncoproteins on the amount of each of the RIPOR2 transcriptional variants. 

**Figure 8:**
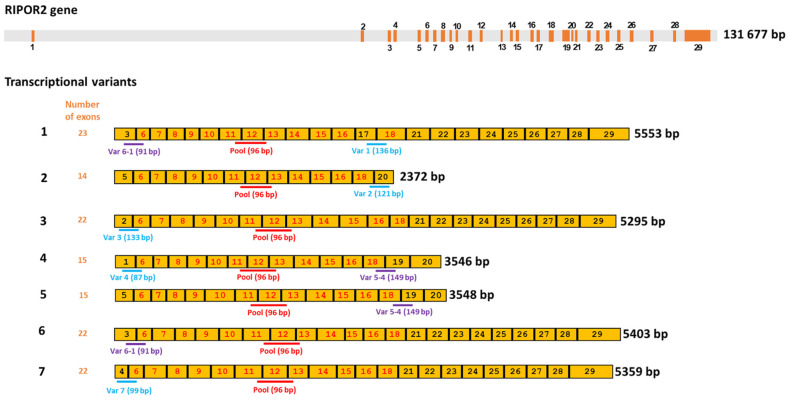
Human RIPOR2 gene and its transcriptional variants. Upper grey bar shows the position of introns in RIPOR2 gene, whereas the enumerated orange boxes, the exons of the gene. Seven transcriptional variants are enlisted under the gene in yellow color, showing the enumeration of the exons that comprise each transcript (1–7). Exons with numbers in red color are those shared by all the transcripts. Below the representation of each the transcriptional variant, the position of the specific primers and the size of the expected amplicon is shown. The pool primers amplify a fragment (shown in red) within a common region. Exons with the numbers in black color are only shared by some transcripts; therefore, primers for specific variants were designed within these areas. Amplicons shown in blue are those that allow the detection of a given specific variant, whereas amplicons in purple are shared by two variants (i.e., variants 1 and 6; variants 4 and 5).

Since few information is available about transcriptional variants of RIPOR2 [37,38], and the primers first used for quantification of RIPOR2 detected all the transcriptional variants (Figure 8), we designed primers to detect the 7 RIPOR2 transcriptional variants (Supplementary Table S1). Due to the similarity among some of the RIPOR2 variants sequences, it was only possible to use specific primers for variants 1, 2, 3, 4, and 7. There are no unique sequences within the exons or in exon–exon junctions distinguishing variants 5 and 6; nevertheless, new primers able to detect variant 5 (and also to detect variant 4), as well as primers detecting variant 6 (and also variant 1) were used. Figure 8 depicts this strategy.

**Figure 9:**
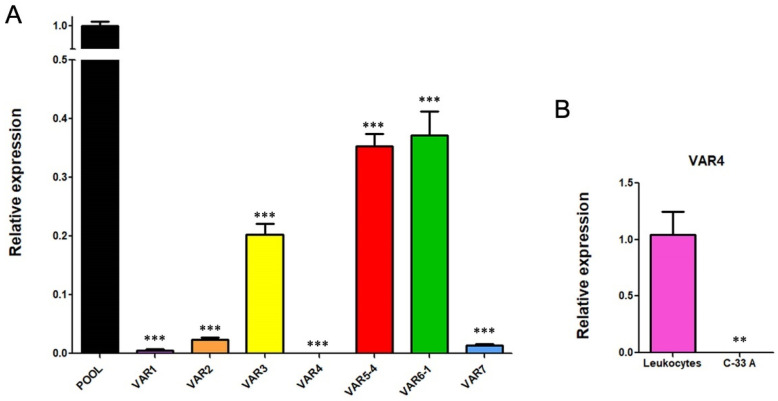
RIPOR2 variants expressed in C-33 A cells. (**A**) RT-qPCR showing the basal levels of the transcriptional variants, compared to RIPOR2-pool levels. (**B**) Expression of variant 4 in lymphocytes compared to C-33 A cells. *** *p* < 0.0001, ** *p* = 0.0066.

To determine the basal gene expression levels of the seven transcriptional variants of RIPOR2 in C-33 A cells, RT-qPCRs were performed. Figure 9A,B show the expression of each transcriptional variant compared to the levels of total RIPOR2 transcripts detected by RIPOR2-pool primers. Variants 5 and 6 were the most abundant in relation to the other variants, which were reduced in 2.84- and 2.68-fold respectively, followed by variant 3 with a reduction of 4.95-fold, compared with the pool primers. Otherwise, variants 1, 2, and 7 exhibited the lowest levels in this cell line, being decreased 200-, 43.47-, and 76.9-fold, respectively. Interestingly, we could not detect variant 4 in C-33 A cells; nevertheless, we did detect it in human leukocytes (Figure 9C), demonstrating that the primers correctly amplify the variant 4 fragment. 

**Figure 10:**
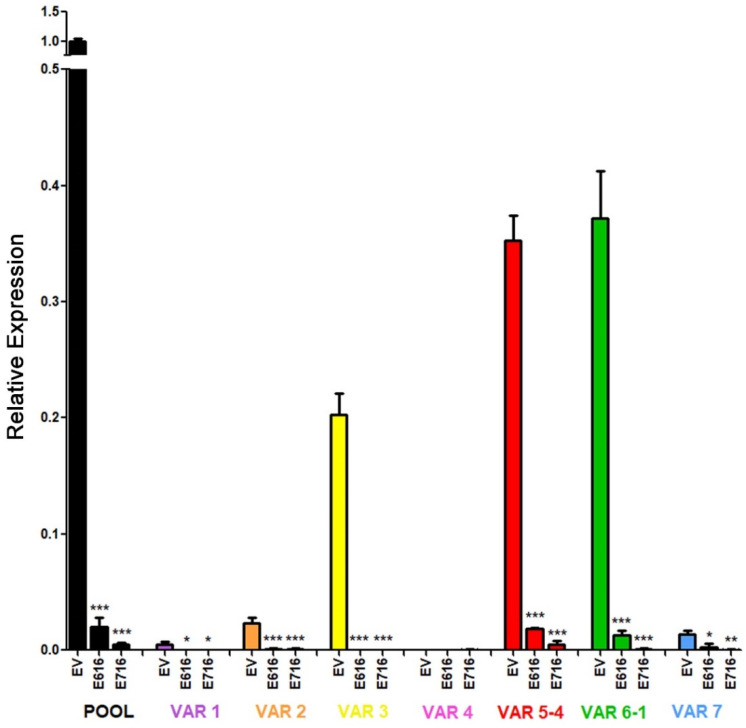
Effect of E616 and E716 oncoproteins on the amount of RIPOR2 transcriptional variants. Expression levels of the 1–7 transcriptional variants were assessed in C33-EV, C33-E616, and C33-E716 cells by RT-qPCR using RIPOR2 pool or specific variant primers. Statistical differences are expressed as *** *p* ≤ 0.0009, ** *p* = 0.0029, and * *p* ≤ 0.0166 when comparing EV vs. E616 or E716 groups.

Further, we investigated the effect of E6 and E7 proteins on mRNA levels of the seven RIPOR2 variants in C-33 A stable transfected cells (Figure 10). When analyzing the expression of all the RIPOR2 transcripts detected with the pool primers, a dramatic decrease in RIPOR2 expression in the presence of E6 and E7 of 50- and 250-fold, respectively, was observed in relation to the EV control. An evident effect of both oncoproteins in the decreased levels of variants 1, 2, 3, 5, 6, and 7 was observed even when the basal expression of some of these variants was low in comparison with the value observed in EV cells. Notably, those variants with the highest expression, such as variants 5 and 6, reduced 20- and 29.4-fold, respectively, in E6-expressing cells, while, in those cells with E7, in 76.9- and 500-fold, respectively. While variant 3 was completely ablated by the viral oncoproteins. As expected, the expression of variant 4 was not detected in all tested groups.

**Figure 11:**
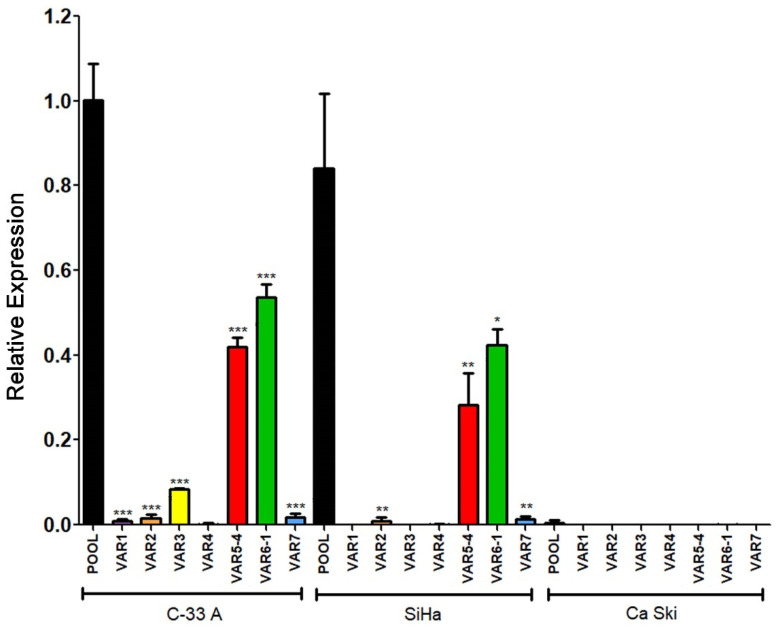
Expression of RIPOR2 transcriptional variants in cervical cancer cell lines. The levels of the 7 transcriptional variants were evaluated in C-33 A, SiHa, and Ca Ski cell lines by RT-qPCR. Fold change data were calculated compared with RIPOR2 pool levels within each cell line, and statistical analyses were performed using GraphPad prism and expressed in mean ± SD, significance is represented as *** *p* ≤ 0.009, ** *p* ≤ 0.0072, and * *p* = 0.0159.

Expression data obtained from C-33 A, SiHa, and Ca Ski cells regarding the seven transcriptional variants analyzed are shown in Figure 11. Interestingly, Variants 5 and 6, the most abundant previously observed in C-33 A (Figure 9) were also the most enriched in SiHa cells. Other variants, such as 2 and 7, were poorly expressed in SiHa cells, while 1, 3, and 4 were absent. Moreover, no expression of any variant was observed in the Ca Ski cell line, correlating with the absence of RIPOR2 pool transcripts observed in Ca Ski cells (Figure 7C). 

### 3.6. RIPOR2 Expression Is Downregulated in Premalignant Lesions and Lower Levels of RIPOR2 Are Associated with Worse Prognosis of Cervical Cancer

**Figure 12:**
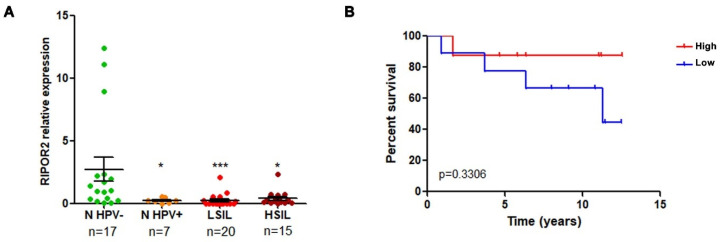
RIPOR2 expression in cervical premalignant lesions and cervical cancer. (**A**) RIPOR2 mRNA levels were analyzed by RT-qPCR using RIPOR2 pool primers in normal (*n*) HPV positive and negative samples, as well as LSIL and HSIL * *p* ≤ 0.0222; *** *p* = 0.0001. (**B**) Overall survival analysis comparing RIPOR2 low (blue line) vs. high (red line) expression in cervical cancer patients (*p* = 0.3306).

RT-qPCR analysis was performed to determine whether the expression of RIPOR2 was altered in premalignant lesions of the cervix comprising low and high grade squamous intraepithelial lesions (LSIL and HSIL) and normal samples with HPV infection, compared to normal HPV negative samples. As shown in Figure 12A, the expression of RIPOR2 significantly decrease as the cervical lesion progresses. In addition, the evaluation of RIPOR2 expression in cervical cancer cases (*n* = 19) showed that the low expression of RIPOR2 was associated with a worse OS, although no significant results were obtained, probably due to the lack of an adequate number of samples available; therefore, a larger cohort of CC samples is required to ascertain this association (Figure 12B). 

Taken together, these results suggest that RIPOR2 expression is downregulated by HPV-16 E6 and E7 oncoproteins, and it is probably affected from the onset of infection; moreover, our data indicate that decreased expression of RIPOR2 is associated with unfavorable clinical outcome of patients.

## 4. Discussion

In Mexico, cervical cancer continues to be an important health problem, where a vast majority of cases are diagnosed in advanced stages [9]. For those patients, conventional treatments may not be as effective, so targeted strategies could give better results. In this sense, the search for prognostic markers becomes an area of interest, to identify patients who may benefit from specific therapies, in addition to identifying possible therapeutic targets.

The continuous expression of HPV E6 and E7 oncoproteins promotes and maintains the malignant phenotype in CC. It has been demonstrated that reducing the expression of E6 and E7 oncogenes of HPV-16 reverses the malignant phenotype. In this regard, an in vivo study revealed that a xenograft HPV positive tumor mice model that was locally injected with liposomes containing a CRISPR-Cas9 knocking-down system for E6/E7 from HPV-18 and -16, recovered the expression of p53 and p21 tumor suppressors, followed by a reduction in tumor growth [39,40]. Additionally, it was recently demonstrated that restoration of p53 expression and inhibition of HPV-16 E7 by CRISPR-Cas9 system delivered in nanoparticles in xenograft mice tumors induces a reduction of tumor growth and it is worth mentioning that such treatment exhibits a low toxicity and high transfection efficiency [41]. However, the use of CRISPR/Cas vectors specifically targeting E6 or E7 in tumor cells is still limited since such vectors have demonstrated low safety and are restricted to a specific HPV viral type. This prompts the study of not only the viral oncoproteins but also their molecular targets that could be used as prognostic biomarkers and/or as pharmacological targets to improve the quality of life of patients with cervical cancer.

Previous efforts have been made to identify molecules that allow predicting the clinical outcome of CC patients, based on deregulated molecules in cancer [42], or on the presence and expression of viral oncogenes [43]. Although little information is available about those cellular elements deregulated by viral oncoproteins that could be used as biomarkers associated with clinical outcome in CC. The study of molecules based on RNAs, identified by massive RNA sequencing, provides extensive information on those molecules altered in cervical cancer [16], in addition to those altered by viral oncogenes that could eventually serve as prognostic biomarkers, as is proposed in this study. With this in mind, we analyzed the transcriptome of cervical cancer C-33 A cells stably transfected with HPV E6 or E7 oncogenes, to further identify potential prognostic biomarkers in CC. This work led to the identification of genes deregulated by both viral oncoproteins that also were found to be altered in CC and associated with overall survival. As a result, we show for the first time that E6 and E7 oncoproteins suppress the expression of RIPOR2 and increases the expression of PFKFB4, which in turn was associated with poor survival in CC patients.

PFKFB4 (Phosphofructo-2-kinase/fructose-2,6-bisphosphatase 4) is one of four isoenzymes of PFKFB [44], which generate fructose-2,6-bisphosphate, an allosteric activator of 6-phosphofructo-1- kinase, which is a rate-limiting enzyme in glycolysis and regulate the pentose phosphate pathway. Recent studies have demonstrated that the high expression of PFKFB4 predicts a poor prognosis in various types of cancer, including breast [45], gastric [46]; lung [47]; melanoma [48]; and thyroid cancer [49]. In this work, the RNAseq analysis revealed that PFKFB4 is overexpressed in the presence of E6 and E7, and the data obtained by qPCR showed a trend towards increased expression of this gene, although not significant; probably because PFBKB4 expression could be highly sensitive to regulation by other cancer-associated processes such as hypoxia [50], which warrants further study.

The family of RIPOR (RHO family interacting cell polarization regulators) proteins comprises 3 isoforms termed RIPOR1, RIPOR2 and RIPOR3, encoded by the FAM65A, FAM65B and FAM65C genes, respectively. RIPOR proteins bind directly to RHO GTPases (A, B, and C) through their RHO-binding motif, thereby inhibiting RHO activity and negatively influencing cellular functions regulated by these GTPases, such as receptor trafficking, cell migration, growth and polarization [51].

There is little information on the involvement of RIPOR2 in cancer. Dakour et al., in 1997 [37], described the lack of expression of RIPOR2 in a wide variety of proliferating cancer cell lines. Tumors derived from prostate cancer cell line PC3, exhibited low expression of RIPOR2, even though a stem-like subpopulation derived from such cell line showed the opposite phenotype [52]. A bioinformatic study revealed that a signature comprising four genes (RIPOR2, DAAM2, SORBS1, CXCL8) was found to be associated with survival in cervical cancer patients [53]. Moreover, those tumors with this signature where RIPOR2 was downexpressed had a worse prognosis. Furthermore, the presence of RIPOR2 in tumors is positively associated with the infiltration of CD8 + cells T cells, macrophages, neutrophils, and dendritic cells. Furthermore, those patients whose tumors express RIPOR2 in the signature exhibit high expression of PD-1, PD-L1, PD-L2, and CTLA-4, making them potential candidates for immune checkpoint inhibitors. 

In agreement, a recent study identified a four gene antitumor signature related to the tumor microenvironment, which included RIPOR2, CCL22, PAMR1, and FBN1 genes [54]. Authors found that tumors with high expression of RIPOR2, had a lower mutation burden, and higher levels of CD8 + T cells. Interestingly, patients with those tumors presented a better response to immunotherapy with antibodies against PD-1 alone or combined with CTLA4. Concordantly with the results obtained in the present work, the authors found that CC patients with higher RIPOR2 expression had a longer overall survival, concluding that RIPOR2 is a protective factor in CC. Moreover, when RIPOR2 was overexpressed in SiHa and HeLa CC cell lines, cell viability and migration capacity significantly diminished, suggesting that RIPOR2 is a tumor suppressor gene in cervical cancer. In this sense, our work provides valuable information on the participation of E6 and E7 viral oncoproteins in the regulation of RIPOR2 and its association with clinical evolution in CC, regardless of the tumor microenvironment.

It is known that the expression of RIPOR2, which negatively regulates the activation of RhoA GTPase, is promoted by transcriptional factors such as FOXO1 [55]. Previous studies have shown that FOXO1 expression is ablated in cervical tumors compared to normal tissue, and that FOXO1 expression decreases as precancerous lesions progress [56]. However, other studies evidence a controversy on the possible role of FOXO1 in cervical cancer, since its overexpression has been associated with a poor prognosis [57]. Moreover, it has been shown that the inhibition of the expression of E6 and E7 in Ca Ski cells recovers the expression of FOXO1, leading to apoptosis and to a reduction in the proliferation of cancer cells [58]. Interestingly, our RNAseq data showed a decrease in FOXO1 expression of −0.49 and −0.41 log2FC in cells with E6 and E7, respectively (Tables S2 and S3). On the other hand, it has been described that the overexpression of GTPase RhoA in cervical cancer is associated with distant metastasis after concomitant treatment with chemotherapy and radiotherapy [59]; concordantly, it is known that E6 and E7 oncoproteins regulate the activation of the GTPase RhoA [60,61]. This suggests the existence of a FOXO1/RIPOR2/RhoA axis mediated by HPV oncoproteins which is affected in cervical cancer and related to an unfavorable clinical outcome. 

Over time, different names have been used for RIPOR2 (PL48, C6orf32, FAM65B) and it has also been reported with different nucleotide numbers or protein sizes. The first RIPOR2 variants identified in the differentiating cytotrophoblast included three mRNAs (2.8, 3.5 and 4.8 kb) [37,62]. Subsequently, multiple isoforms of the RIPOR2 protein were detected by immunoblot, and those described as isoforms 1 and 2, which were composed of 1018 and 591 amino acids, respectively [38], correspond to isoforms 6 and 2 of the protein according to most recent NCBI data [36] (Table 2). Furthermore, PL48 was described as a short isoform of C6orf32 composed of 536 amino acids [38], which could be the current variant 2. Therefore, the specific roles of each RIPOR2 isoform in physiological and cancer-related are not yet known.

Our results show a significant decrease in the expression of the transcriptional variants of RIPOR2 by the E6 and E7 oncoproteins, which suggests that their modulation is at the transcriptional level. It is not ruled out that the low expression of RIPOR2 in cells harboring HPV-16 E6 and E7 oncoproteins could involve epigenetic changes in the RIPOR2 promoter, since E6 and E7 oncoproteins have been shown to promote the hypermethylation of various tumor suppressor genes, which is associated with increased cell proliferation [63]. 

Interestingly, according to the Eukaryotic Promoter Database (EPD) [64], four promoters mediating the transcription of RIPOR2 are described. Besides, data derived from the FAMTOM5 project [65], show that expression of the RIPOR2 transcripts from promoters 1, 2 and 4 is decreased in CC cell lines naturally infected with HPV-16, -18 or -68, compared to normal cervical epithelium. Functional analysis of the promoters that regulate the expression of the RIPOR2 transcriptional variants is necessary to elucidate the specific processes involved and the factors participating in these regulations. 

In addition to showing the possible use of RIPOR2 as a prognostic biomarker deregulated by both viral oncoproteins, our study provides information on the molecular mechanisms involved in the establishment and maintenance of tumors with papillomavirus infection and on molecules that could eventually be useful as therapeutic targets. It is important to mention, that also the deregulated molecules identified as dependent on the clinical stage in the multivariate analysis (Table 1), could provide valuable information for therapeutics, even when they do not offer an advantage in prognosis.

Although the present work focuses on the genes that were altered by both oncoproteins, all the genes that were found to be significantly upregulated or downregulated by each of the oncoproteins independently, are of interest to be studied both at the molecular level, as well as for their association with cancer and with the clinical outcome of patients either in TCGA databases or in other cohorts.

It is worth noting that enrichment analysis of Differentially Expressed Genes (DEG) demonstrated that E6 and E7, affect biological functions or pathways related to cancer. For instance, E6 alters glycolysis, translation initiation, carbon metabolism and ROBO-Slit signaling, among others; while E7 affects extra cellular matrix organization, MAPK signaling pathway and focal adhesion pathways, among others. It is known that alterations in such processes drive to increased proliferation, migration, or invasion, which are key elements for cancer development. Those processes have been shown to be affected in other types of cancer. For example, disturbed glucose metabolism has been reported in lung cancer cells [66]; aberrant expression of translation initiation factors is a common feature in gastrointestinal, lung, colorectal, breast, and prostate cancers [67]; moreover, alterations in the Slit/ROBO signaling induce malignant transformation in colorectal cancer [68].

The study of the expression of RIPOR2 when cancer is diagnosed could have a potential utility as a prognostic biomarker that allows the appropriate decision on surveillance and therapeutic intervention in patients with low risk of survival. Undoubtedly, the analysis of RIPOR2 offers a promising tool that would help improve the quality of life of patients. A limitation of this study is that the number of patients from the analyzed Mexican cohorts were restricted to the available samples, being mandatory the validation of RIPOR2 expression as a potential biomarker in a larger cohort of premalignant lesions and cervical cancer samples in a representative proportion of the studied population. On the other hand, we could not detect the RIPOR2 protein in tumor samples nor in cell lysates since the available commercial antibodies had poor immunodetection by western blot and immunohistochemistry; therefore, the obtention of more specific antibodies for the detection of RIPOR2 variants would be valuable to evaluate its association with poor OS in cervical cancer patients. 

Our findings firmly position RIPOR2 as a promising prognostic biomarker in cervical cancer and demonstrate the effect of viral oncoproteins in downregulating RIPOR2 transcriptional variants. However, the specific mechanisms by which E6 and E7 downregulate RIPOR2 and their relationship with the development and/or maintenance of cancer is something that deserves further study. 

## Figures and Tables

**Table 1:** Univariate and multivariate analyses of the genes affecting the overall survival.

		Univariate Analysis		Multivariate Analysis	
	Overall Survival	HR (95% CI)	*p*-Value	HR (95% CI)	*p*-Value
SLC4A11	High vs. low expression	2 (1.2–3.5)	**0.0081**	1.42 (0.79–2.55)	0.228
NUP188		2 (1.2–3.4)	**0.0097**	1.10 (0.54–2.23)	0.773
CREM		2 (1.2–3.3)	**0.013**	0.80 (0.40–1.62)	0.55
AP1B1		1.9 (1.1–3.1)	**0.016**	0.99 (0.52–1.88)	0.99
**RIPOR2**		2.4 (1.4–4.1)	**0.0016**	1.80 (1.00–3.25)	**0.048**
**PFKFB4**		0.5 (0.3–0.84)	**0.0085**	0.50 (0.27–0.93)	**0.029**
CC2D1A		1.9 (1.1–3.2)	**0.015**	1.14 (0.56–2.30)	0.704
BICDL1		1.9 (1.1–3.2)	**0.015**	1.16 (0.62–2.15)	0.629
RHOT2		2 (1.2–3.4)	**0.0073**	1.44 (0.74–2.79)	0.278
NBEAL2		1.9 (1.1–3.2)	**0.016**	1.27 (0.69–2.33)	0.436
CPNE7		2.2 (1.3–3.7)	**0.0033**	1.55 (0.83–2.90)	0.165
FARSA		1.9 (1.1–3.2)	**0.013**	1.15 (0.55–2.40)	0.692
SHTN1		2.2 (1.3–3.7)	**0.0033**	1.46 (0.73–2.91)	0.281
Clinical Stage		1.5 (1.2–1.9)	**0.0003**		

Bold denotes a significant *p* value.

**Table 2:** Transcripts and proteins coded by the RIPOR2 gene.

Transcript	Length (nt)	Transcript Type	Protein Isoform	Length (aa)
1	5553	protein coding	1	1068
2	2372	protein coding	2	591
3	5295	protein coding	3	1047
4	3546	protein coding	4	647
5	3548	protein coding	5	613
6	5403	protein coding	6	1018
7	5359	protein coding	6	1018

## Data Availability

Data is contained within the article and supplementary material.

## Supplementary Materials

The following supporting information can be downloaded at: https://www.mdpi.com/article/10.3390/cells11233942/s1, Table S1: Primers used in this work; Table S2: Gene expression profiles of E616 expressing cells; Table S3: Gene expression profiles of E716 expressing cells.
