# Supplementary material for: RIPOR2 Expression Decreased by HPV-16 E6 and E7 Oncoproteins: An Opportunity in the Search for Prognostic Biomarkers in Cervical Cancer
Source: Cells. 2022 Dec 6;11(23):3942. doi: 10.3390/cells11233942 (PMC9740487; doi:10.3390/cells11233942)
Supplement: Supplementary file 1 [file cells-11-03942-s001.zip › Table S1 Primers used in this work.pdf]

cells

MDPI

Table S1. Primers used for the analyses

TARGET	PRIMER NAME	SEQUENCE 5′→ 3′	PRODUCT SIZE
HPV-16 E6	EcoRI-HA-E616 Fwd	GGGGAATTCATACCCATACGATGTTCCAGAT-TACGCTTTTCAGGACCCACAGGAGC	499 bp
	BglII-E616 Rev	GGGAGATCTTTACAGCTGGGTTTCTCTACGTG	
HPV-16 E7	EcoRI-HA-E716-Fwd	GGGGAATTCATACCCATACGATGTTCCAGAT-TACGCTC ATGGAGATACACCTACA	340 bp
	BglII-E716-Rev	GGGAGATCTTTATGGTTTCTGAGAACAGAT	
HPV-16 E6	E616 Fwd	TTTCAGGACCCACAGGAGCGA	130 bp
	E616 Rev	AGTCATATACCTCACGTCGCAGTA	
HPV-16 E7	E716 Fwd	CAAGTGTGACTCTACGCTTCGG	82 bp
	E716 Rev	TGTGCCCATTAACAGGTCTTCCAA	
18S	18S Fwd	AACCCGTTGAACCCCATT	149 bp
	18s Rev	CCATCCAATCGGTAGTAGCG	
PFKFB4	PFKFB4 Fwd	CAACATCGTGCAAGTGAAACTG	111 bp
	PFKFB4 Rev	GACTCGTAGGAGTTCTCATAGCA	
RIPOR2 pool	RIPOR2 pool Fwd	GAGCTTCAAGGAGTACACAGAG	96 bp
	RIPOR2 pool Rev	CCAGCCAGACCTTTCATCTT	
RIPOR2 VAR 1	RIPOR2 VAR 1 Fwd	CTGTCTGTCTTGAGTGCCTTG	136 bp
	RIPOR2 VAR 1 Rev	AGATGTCATCAGGTAGATTTGAATAGAG	
RIPOR2 VAR 2	RIPOR2 VAR 2 Fwd	GGATGATATTCTAAAAAAGTAGAGAAG	121 bp
	RIPOR2 VAR 2 Rev	GAAGTCAGCAGGTTGAAGAATAGG	

RIPOR2 VAR 3	RIPOR2 VAR 3 Fwd	AGTGTGACTGCTGAGACTG	133 bp
	RIPOR2 VAR 3 Rev	GTCGGTAGTCCTTCACCAAA	
RIPOR2 VAR 4	RIPOR2 VAR 4 Fwd	TGGTGTTACCTTCGCGATTAC	87 bp
	RIPOR2 VAR 4 Rev	TGGTCGGTAGTCGGTTGA	
RIPOR2 VAR 5-4	RIPOR2 VAR 5-4 Fwd	GGATGATATTCTAAAAGATGCTAAACAC	149 bp
	RIPOR2 VAR 5-4 Rev	ACTCAAGATGGCACAAAAGC	
RIPOR2 VAR 6-1	RIPOR2 VAR 6-1 Fwd	ATTGGTGCGGAGGCTTT	91 bp
	RIPOR2 VAR 6-1 Rev	GAGTCTGGTCGGTAGTCCTAA	
RIPOR2 VAR 7	RIPOR2 VAR 7 Fwd	GGTACGGTCGGGAAGTTG	99 bp
	RIPOR2 VAR 7 Rev	GTAGTCCTTGGCCCGTTC	
